# Effects of Nitrogen on Microstructure and Properties of SDSS 2507 Weld Joints by Gas Focusing Plasma Arc Welding

**DOI:** 10.3390/ma17215375

**Published:** 2024-11-03

**Authors:** Tianqing Li, Kai Wang, Yucheng Lei

**Affiliations:** School of Materials Science and Engineering, Jiangsu University, Zhenjiang,212013, China

**Keywords:** super duplex stainless steel, plasma arc welding, nitrogen, microstructure, austenite phase

## Abstract

Regulating the phase ratio between austenite and ferrite in welded joints is crucial for welding super duplex stainless steel. Nitrogen plays a significant role in maintaining an optimal phase ratio. In this study, the focusing gas channel of gas-focused plasma arc welding was utilized to introduce nitrogen into the arc plasma, which was then transferred to the weld pool. Experiments with and without nitrogen addition were designed and conducted to examine the effects of nitrogen on the microstructure and properties of SDSS 2507 weld joints. The results show that nitrogen addition increased the austenite content in the weld metal from 22.2% to 40.2%. Nitrogen also altered the microstructure of the austenite, changing it from thin grain boundary austenite and small intragranular austenite to a large volume of coarse, side-plate Widmanstätten austenite. The ferrite in the weld metal exhibited a preferred orientation during growth, while the austenite showed a disordered orientation. Additionally, the maximum texture intensity of the ferrite decreased with nitrogen addition. Nitrogen addition led to an increase in the microhardness of the austenite in the weld metal, attributed to the solid solution strengthening effect of nitrogen and increased dislocation tangling, while it decreased the microhardness of the ferrite. This study enhances the welding theory of 2507 super duplex stainless steel and guides the practical application of gas-focused plasma arc welding for 2507 super duplex stainless steel.

## 1. Introduction

Duplex stainless steel (DSS) and super duplex stainless steel (SDSS) possess high strength and excellent corrosion resistance [1,2,3], and therefore, they have been widely applied in various environments with high pressure and heavy corrosiveness, such as marine, chemical industries, offshore oil and gas industries. The superior performance arises from the special microstructure composed of a near equal volume fraction of ferrite phase (α) and austenite phase (γ). However, the ferrite content may increase substantially and undesired precipitates may form in the weld metal (WM) and heat-affected zone (HAZ) due to the rapid cooling rate and the loss of nitrogen during welding [4,5,6,7], in which case the mechanical property and corrosion resistance of welded joints may be destroyed [8,9]. Various traditional fusion welding methods, such as gas tungsten arc welding (GTAW), shielded metal arc welding (SMAW), gas metal arc welding (GMAW), flux-cored arc welding (FCAW), etc., have been used for the welding of DSS and SDSS to solve these problems recently [10,11,12,13]. Gupta A et al. [10] found that the ferrite content of the WM of SDSS 2507 decreased with the increase of the heat input using SMAW with an ER2595 electrode. Kim S-T et al. [11] compared the microstructure and corrosion resistance of SDSS weld joints by GTAW with pure Ar and Ar + 5%N_2_ as the shielding gas and found that the addition of nitrogen increased the content of the austenite phase. The results also showed that more Cr_2_N precipitated in the HAZ than that in the WM and the content of Cr_2_N in the WM decreased due to the nitrogen addition, resulting in the increase of the resistance against pitting corrosion. Similar results were obtained by Zhang Z et al. [12]. The weld joints of UNS S31803 DSS were fabricated by GTAW and FCAW with Ar + 2%N_2_ as the shielding gas. It was found that nitrogen increased the austenite content and reduced the tendency for Cr_2_N precipitation of the WM and HAZ, contributing to the improvement of corrosion resistance and impact toughness. The pitting degree of the HAZ was more severe than the WM because of the greater precipitation of Cr_2_N. The previous works indicated that the conventional fusion welding process may obtain WM with an ideally proportional two-phase by controlling the heat input or adding alloying elements during welding. However, these methods inevitably present some disadvantages, such as poor welding speed and efficiency, large distortion, low penetration depth/width ratio and wide size of the HAZ, which may become the area prone to pitting corrosion.

Therefore, high-energy-density welding methods such as laser beam welding (LBW), electron beam welding (EBW) and plasma arc welding (PAW) have attracted increasing attention for the welding of DSS and SDSS recently owing to the high welding speed and efficiency, high penetration depth/width ratio, and narrow fusion zone (FZ) and HAZ [14,15]. Lai R et al. [16] studied the effect of nitrogen in the shielding gas on the microstructure and corrosion resistance of DSS 2205 welded joint using LBW. The result showed that the addition of nitrogen not only improved the austenite content and the corrosion resistance significantly but also affected the austenite morphology. Similar experiments were carried out by Muthupandi V et al. [17]. They introduced nickel and nitrogen into the WM of UNS 31803 DSS by LBW and EBW and found that the nitrogen and nickel effectively decreased the ferrite content in the WM. Zhang Z et al. [18] studied the influence of heat input on the microstructure and properties of UNS 31803 DSS welded joint by EBW. It was found that the increase of the heat input had little effect on the increase of the austenite content and pitting corrosion resistance and the decrease of the ferrite texture’s strength and hardness. The change in the heat input did not restrain the precipitation of Cr_2_N. The toughness and microstructural of SDSS 2507 WM with two different heat inputs were studied by Taban E et al. [15] using PAW. The results showed that the ferrite content just decreased by 5% with the increase of the heat input and two welded joints exhibited good low-temperature impact toughness. Migiakis K et al. [19] studied the effect of the alloying element on the microstructure of UNS S32760 SDSS weld joints by the plasma transferred arc (PTA) technique, where Ar + N_2_ was used as the shielding gas and plasma gas and high Ni content filler metal were used. The researchers found that the addition of nitrogen and nickel strongly promoted the formation of the austenite phase. According to the previous work, it is not difficult to summarize that for high-energy-density welding methods with rapid heating and cooling rates, the influence of the alloying elements (Ni or N) on the microstructure and properties of an SDSS weld joint is much stronger than that of the heat input, as found by Muthupandi V et al. [20].

How to regulate the phase ratio in welded joints is the key to the welding of super duplex stainless steel. Adding nitrogen to the weld is a good method to improve the austenite phase ratio. In this work, the focusing gas channel of gas focusing plasma arc welding is used to transport nitrogen to the arc plasma. Gas focusing plasma arc welding is a new modified plasma arc welding method. Does the nitrogen, as the focusing gas, increase the austenite phase ratio? This study will investigate the microstructure and properties of SDSS 2507 welded joints by gas focusing plasma arc welding. Determining the effects of nitrogen on SDSS 2507 weld joints by gas focusing plasma arc welding is one of the urgent needs. Therefore, this work will focus on this topic. Firstly, this study will design gas focusing plasma arc welding experiments with and without nitrogen addition. Then, the optical microscope (OM) and electron backscatter diffraction (EBSD) will be used to characterize the microstructure. X-ray diffraction (XRD) will be used to characterize the crystalline structure. TEM will be used to characterize the dislocation. Also, the microhardness and pitting corrosion resistance of SDSS 2507 weld joints will be measured. Finally, the effects of nitrogen on the microstructure and properties of SDSS 2507 weld joints by gas focusing plasma arc welding will be discussed. This study will enrich the theory of super duplex stainless steel by gas focusing plasma arc welding and provide guidance for practical welding applications on super duplex stainless steel 2507.

The nomenclature used in this work is shown in Table 1.

## 2. Materials and Experimental Procedure

**Figure 1:**
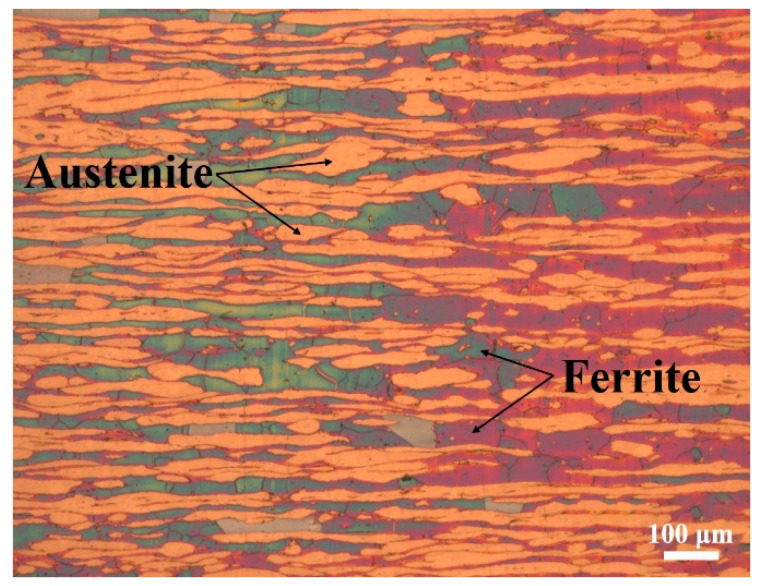
Microstructure of SDSS 2507.

Super duplex stainless steel (SDSS 2507) was chosen as the base material in this work. The chemical composition of SDSS 2507 is shown in Table 2. The microstructure of SDSS 2507 is composed of a ferrite phase and an austenite phase with an approximately equal volume fraction, as observed in Figure 1.

**Figure 2:**
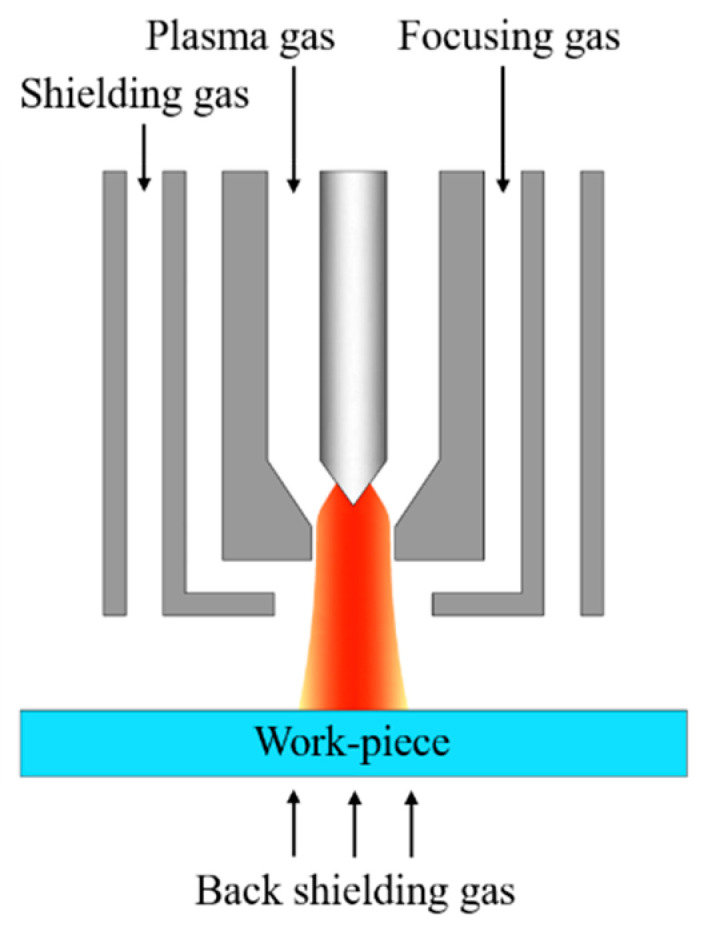
Internal structure of the GF-PAW welding torch.

Gas focusing plasma arc welding, shortened as GF-PAW, was applied to weld the base metal SDSS 2507, which was 80 mm × 40 mm × 2 mm in size. The plate was rolled. The samples were polished mechanically by an angle grinder with a grinding disc and then cleaned with absolute ethanol before welding. The welding direction was perpendicular to the rolling direction (RD) and parallel to the transverse direction (TD) of the base metal. Figure 2 shows the internal structure of the welding torch for GF-PAW. Compared to the conventional PAW welding torch, a focusing gas channel was added between the plasma gas channel and the shielding gas channel in the GF-PAW welding torch. In this work, nitrogen with a purity of 99.99% was used as the focusing gas. Argon with a purity of 99.99% was used as the plasma gas, shielding gas and back shielding gas in this study. Considering the significant effect of nitrogen on the microstructure of the weld, the flow rate of the focusing gas was accurately adjusted by gas mass transfer controller. In order to explore the effect of nitrogen addition on weld joints, experiment A and experiment B were carried out. The focusing gas flow rate was 0 L/min in experiment A and 0.11 L/min in experiment B, respectively. Other welding parameters are shown in Table 3.

The weld joints were polished with the metallographic polishing method and then etched with Beraha Ⅱ solution (60 mL H_2_O + 30 mL HCL + 1 g K_2_S_2_O_5_) for approximately 12 s. The microstructures of the WM, HAZ and base metal (BM) were observed by optical microstructure (OM). ImageJ V1.8.0 software was used to analyze the OM picture. The phase composition of the weld joints was analyzed by X-ray diffraction (XRD). The angle range for scanning was set from 20° to 90°, and a step size of 0.2° was chosen. Jade V6.5 software was used to analyze the XRD data. In order to analyze the volume fraction and the crystallographic orientation information of the two phases in the WM, specimens for experiment A and experiment B on the RD-TD plane were prepared for electron backscatter diffraction (EBSD) analysis. CHANNEL5 V5.12 software was used to analyze the EBSD data. The distribution of elements in the two phases at different zones was analyzed by electron probe microanalysis (EPMA). Transmission electron microscopy (TEM) was used to observe the dislocation in the WM. The microhardness of different zones was measured under a load of 300 gf for a dwell time of 15 s. The microhardness of the ferrite phase and austenite phase in the WM was measured, respectively, under a load of 50 gf for a dwell time of 15 s. Hardness measurements were taken in at least five different locations within each phase in the weld and base material, and the average of these measurements was used as the mean hardness value. Origin software was used to plot the figures.

## 3. Results and Discussion

### 3.1. Effect of Nitrogen on Microstructure and Element Distribution

**Figure 3:**
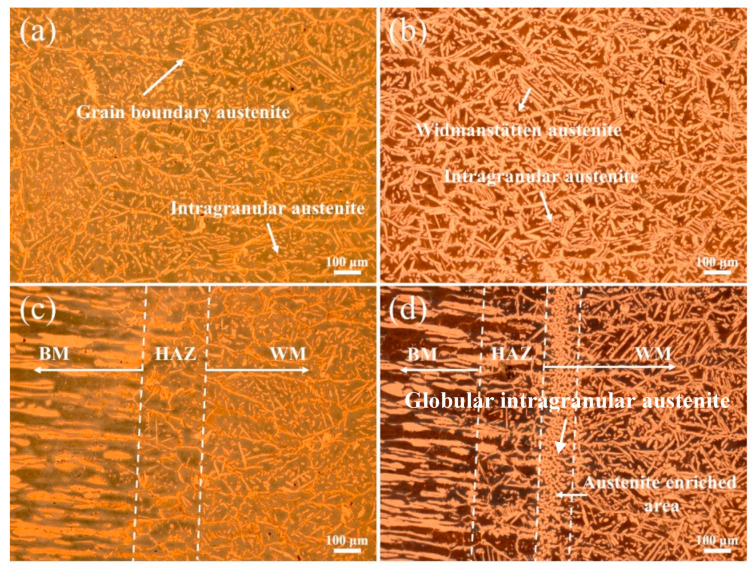
Optical microstructures of weld joints: (**a**) WM in experiment A, (**b**) WM in experiment B, (**c**) HAZ in experiment A, and (**d**) HAZ in experiment B.

The optical microstructures of the WM and HAZ in experiments A and B are shown in Figure 3. The addition of nitrogen significantly increased the volume fraction of austenite and changed the microstructure of austenite. In the WM in experiment A, austenite mainly existed in the morphology of thin grain boundary austenite and small intragranular austenite, with a little Widmanstätten austenite (Figure 3a). In the WM in experiment B, the grain boundary austenite was not obvious, the amount of intragranular austenite decreased and a great quantity of side-plate Widmanstätten austenite appeared (Figure 3b). The addition of nitrogen had no significant effect on the microstructure of the HAZ (Figure 3c,d), which was composed of excessive ferrite and a little grain boundary austenite and intragranular austenite. It is worth noting that significant austenite enrichment occurred near the fusion line. Generally, the solidification mode of SDSS is ferrite mode: ferrite first forms from liquid metal during high temperature cooling, and then austenite forms from the solid-state phase transition of ferrite to austenite in the order of grain boundary austenite, Widmanstätten austenite and intragranular austenite [12,20]. Grain boundary austenite firstly nucleates and grows at the boundaries of ferrite. If enough cooling time is allowed, side-plate Widmanstätten austenite may form from grain boundary austenite and then grow inside ferrite on one side. Compared with the other two forms of austenite, intragranular austenite requires a greater nucleation driving force and undercooling and thus always nucleates in the ferrite grain at a lower temperature [20]. Therefore, abundant intragranular austenite appeared in experiment A due to the rapid cooling rate (Figure 3a). The addition of nitrogen changed the nucleation and growth of the austenite in the WM. The influence mechanism of nitrogen on the austenite in the WM of SDSS using GF-PAW can be explained from four aspects. (1) As one of the strongest austenite-forming alloying elements, nitrogen can significantly increase the volume fraction of austenite. (2) The addition of nitrogen raises the initial nucleation temperature of austenite [17]. (3) As an interstitial element, nitrogen has a greater diffusion rate, resulting in faster nucleation and growth of austenite. (4) The addition of focusing gas provides higher welding energy [21], which results in a lower cooling rate, thus more time is allowed for the ferrite to austenite transformation. Therefore, when nitrogen was added, more intragranular austenite nucleated and then grew together with Widmanstätten austenite at a higher temperature to form coarse, side-plate Widmanstätten austenite (Figure 3b). Abundant globular intragranular austenite appeared in the austenite-enriched area (Figure 3d) because the concentration of nitrogen atoms was the highest in this area.

**Figure 4:**
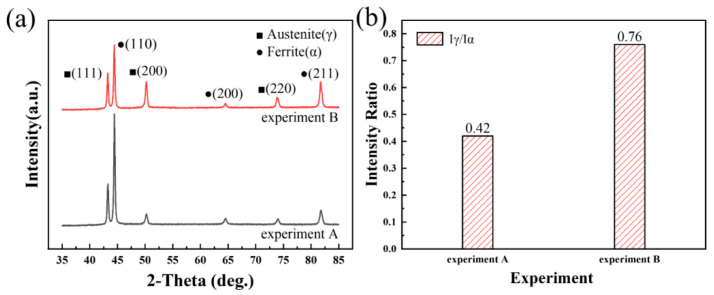
Influence of nitrogen on the XRD patterns of weld joints: (**a**) XRD patterns, and (**b**) ratio of the total peak intensity of austenite to ferrite.

Figure 4 illustrates the impact of nitrogen addition on the X-ray diffraction (XRD) patterns of the weld joints, highlighting its influence on the phase composition. The microstructure of the two weld joints analyzed was primarily composed of ferrite and austenite phases, typical of duplex stainless steel welds. These two phases exhibit distinct crystal orientations and diffraction peaks due to their different atomic arrangements. During the solidification process within the weld metal (WM), the ferrite and austenite phases grow preferentially along specific crystallographic planes. As shown in Figure 4a, the majority of ferrite crystals align and grow along the (110)_ferrite_ close-packed plane, while austenite crystals predominantly grow along the (111)_austenite_ close-packed plane. These orientations contribute to the distinct diffraction peaks seen in the XRD patterns, allowing each phase to be quantitatively and qualitatively analyzed. Generally, the relative content of each phase within the weld joint can be estimated by comparing the intensities of these corresponding diffraction peaks. Figure 4b presents the calculated ratio of the total peak intensities of austenite to ferrite, demonstrating that the addition of nitrogen significantly increases this ratio. This increase indicates a higher proportion of the austenite phase in the nitrogen-added welds, suggesting that nitrogen effectively promotes austenite formation. This enhancement of the austenite content is beneficial, as it can improve the mechanical properties and corrosion resistance, which are critical in duplex stainless steels used in aggressive environments.

**Figure 5:**
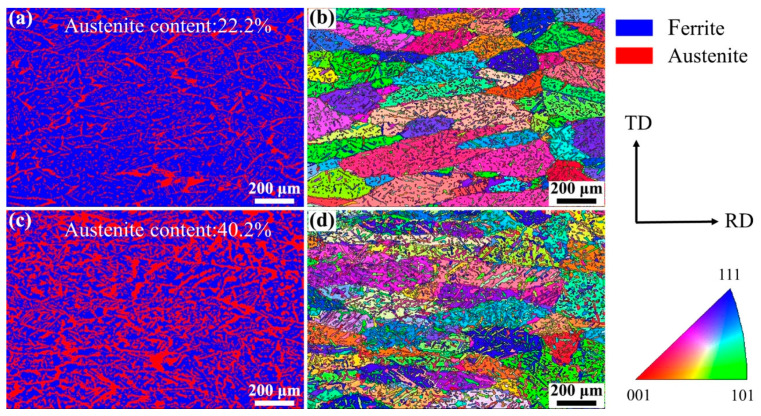
EBSD images of the WM: (**a**) phase map of experiment A, (**b**) IPF of experiment A, (**c**) phase map of experiment B, and (**d**) IPF of experiment B.

**Figure 6:**
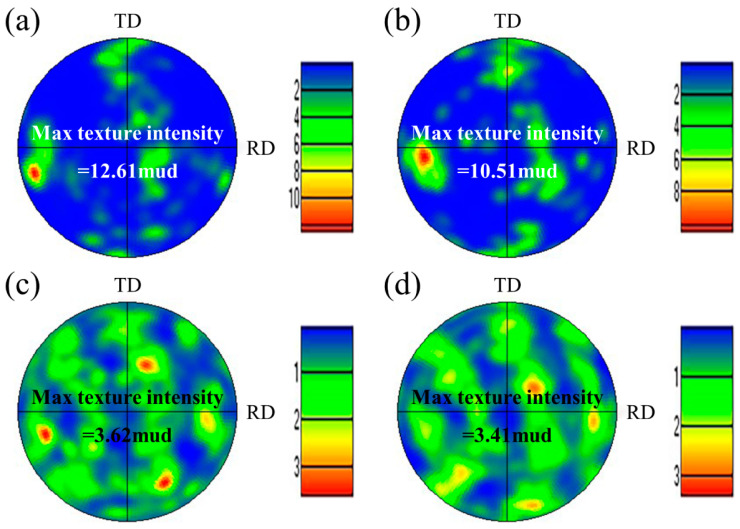
(001) PFs of two phases in the WM: (**a**) ferrite in experiment A, (**b**) ferrite in experiment B, (**c**) austenite in experiment A, and (**d**) austenite in experiment B.

Figure 5 shows the EBSD images of the WM in experiments A and B. The austenite content can be calculated from the phase maps (Figure 5a,c): 22.2% in experiment A and 40.2% in experiment B. A distinctive feature is found in that the small count addition of nitrogen (0.11 L/min) increased the content of austenite in the WM from 22.2% to 40.2% obviously. The microstructure and the crystallographic orientation information of the two phases can be analyzed from the inverse pole figure (IPF). The ferrite grains existed in the form of columnar crystals and the addition of nitrogen did not change the microstructure of the ferrite grains, as shown in Figure 5b,d. One single ferrite grain showed one single orientation, while the austenite grains in one ferrite grain and in different ferrite grains showed different orientations, indicating that the crystallographic orientation of austenite was weaker than that of ferrite. The crystallographic orientation information can be analyzed more intuitively by the pole figure (PF). The ferrite phase in the WM in experiments A and B all showed strong texture in the RD direction on the RD–TD plane (Figure 6a,b). In other words, the preferred orientation direction of the ferrite phase was roughly parallel to the RD of the BM, indicating that the addition of nitrogen did not significantly change the preferred orientation direction of ferrite. The reason for this may be that when the molten pool solidifies, the ferrite grains nucleate from the fusion boundary and then grow toward the weld center to form a large number of columnar crystals, which is controlled by the thermal gradient. However, it should be noted that the max texture intensity of the ferrite phase in the WM in experiment A was stronger than that in experiment B. According to the analysis of the optical microstructure, the reason for this may be that the Widmanstätten austenite grows together with intragranular austenite from one side of the ferrite grain boundary to the other side; thus, the Widmanstätten austenite separates a whole piece of ferrite and weakens the texture intensity of ferrite. Compared with the ferrite phase, the orientation of the austenite phase was disordered, and the max texture intensity was lower (Figure 6c,d) because austenite precipitated and grew randomly from the ferrite grain boundary and inside the ferrite grain.

The effect of nitrogen on the content of the alloying elements (Cr, Ni, Mo, N) in ferrite and austenite in different zones can be analyzed from Table 3. In the BM, Cr and Mo were concentrated in the ferrite phase, while Ni and N were concentrated in the austenite phase [22]. The partitioning of the alloying elements in the WM is different from that in the BM. Although there were some unavoidable nitrogen losses, most nitrogen dissolved in austenite due to the low content of austenite; thus, the N content in the austenite in the WM in experiment A was higher than that in the BM. The addition of nitrogen suppressed the nitrogen loss and increased the austenite content. The high austenite content resulted in the dilution of the nitrogen content in each austenite grain; thus, the N content in the austenite in the WM in experiment B was lower than that in experiment A. The N content in the austenite-enriched area was significantly higher than that in the interior of the WM. The addition of nitrogen significantly promoted the partitioning of Ni and N in austenite and Cr and Mo in ferrite, indicating that the segregation degree of the alloying elements in the WM can be reduced by increasing the N content [18]. The partitioning of the alloying elements in the HAZ was similar to that in the BM, where the addition of nitrogen showed little influence on the content of the alloying elements.

Generally, the factors affecting the pitting corrosion resistance of SDSS include the ratio of austenite to ferrite, the value of the pitting resistance equivalent number (PREN) of each phase and the precipitation and quantity of chromium nitrides (Cr_2_N) in ferrite [23,24,25,26,27]. Silva and Rovere found that the main cause of pitting resistance loss is spinodal decomposition [28]. Due to various constraints, this study focused only on the effect of the PREN on pitting corrosion. The PREN can be calculated according to the following formula: PREN = wt.%Cr + 3.3 wt.%Mo + 16 wt.%N. The PREN can be used to evaluate the pitting corrosion resistance of each phase. The higher the PREN, the better pitting corrosion resistance. The resistance to pitting corrosion of SDSS is generally evaluated by the lower PREN of the two phases. The PRENs of ferrite and austenite in different zones are listed in Table 4. The PREN of ferrite is obviously lower than that of austenite, and thus, the pits may mostly occur in ferrite. A continuous network of austenite with a high PREN can limit the propagation of pitting corrosion in ferrite [29,30]. The addition of nitrogen increases the density of the austenite network; thus, the quantity of pits in the WM in experiment B will be less than that in experiment A.

### 3.2. Effect of Nitrogen on Microhardness

**Figure 7:**
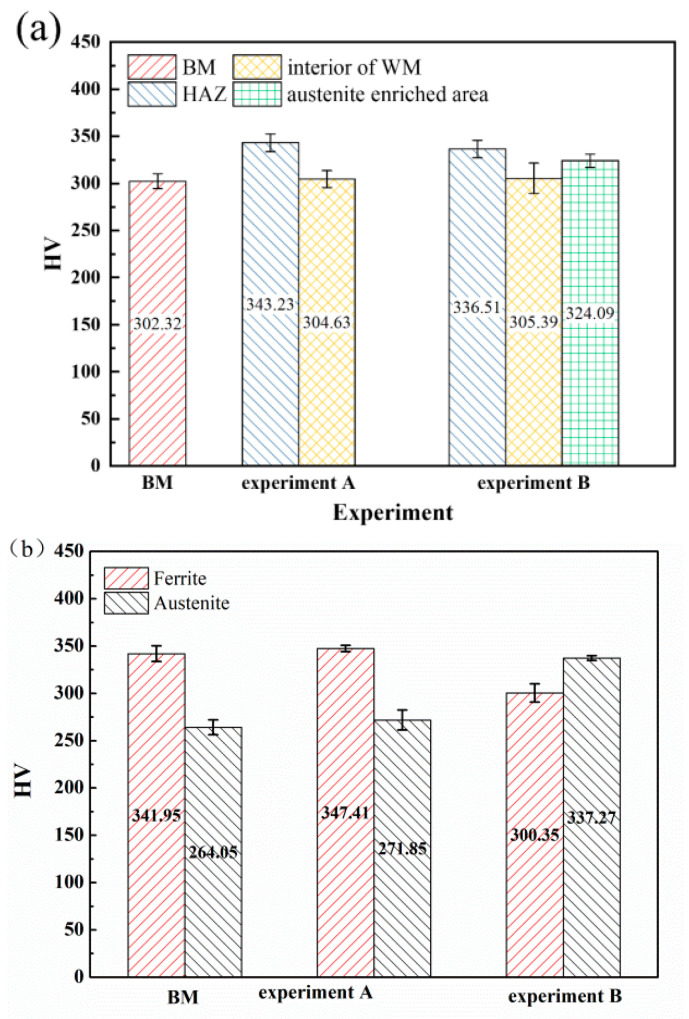
The microhardness of the weld joints and two phases in experiments A and B: (**a**) different zones of the weld joints, and (**b**) two phases in the WM.

**Figure 8:**
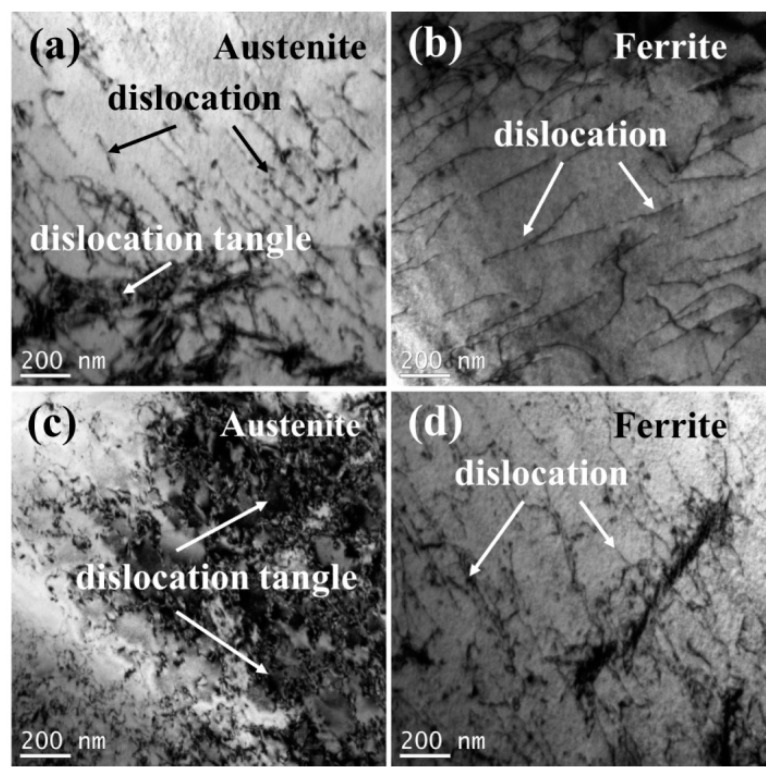
The dislocation characterization of the two phases in the WM: (**a**) austenite in experiment A, (**b**) ferrite in experiment A, (**c**) austenite in experiment B, and (**d**) ferrite in experiment B.

The microhardness of different zones of the weld joints and two phases in the WM in experiments A and B are shown in Figure 7. The HAZs all exhibited the highest microhardness in experiments A and B due to the coarse grains and high content of the ferrite phase, which is generally identified as the strengthening phase in duplex stainless steel [31]. The BM exhibited the lowest microhardness. The addition of nitrogen showed little influence on the microhardness of the interior of the WM. The microhardness of the austenite-enriched area was higher than that of the interior of the WM (Figure 7a). Generally, the microhardness of duplex stainless steel may rise with the increase of the ferrite content [32,33]. In order to further explore the effect of nitrogen on the microhardness of the WM, the microhardness (Figure 7b) and the dislocation characterization (Figure 8) of the ferrite and austenite in the WM were analyzed. Compared with the BM, the microhardness of the two phases in the WM in experiment A increased. The reason for this may be solid solution strengthening [34] caused by nitrogen segregation in austenite (Table 3). An interesting phenomenon was observed: in the welds with nitrogen addition, the hardness of the austenite phase is greater than that of the ferrite phase. In contrast, in the base material and in the welds without nitrogen addition, the hardness of austenite is lower than that of ferrite. The austenite phase is generally identified as the ductile phase in SDSS [35]; thus, compared with the ferrite phase, there may be more dislocation multiplication and dislocation tangle (Figure 8), which can increase the microhardness of the microstructure. The addition of nitrogen significantly increased the amount of dislocation tangle in the austenite. Thus, there may be two factors that increase the microhardness of austenite: (a) solid solution strengthening of nitrogen; and (b) more dislocation multiplication and dislocation tangle. The reduction of the chromium content may decrease the microhardness of ferrite.

## 4. Conclusions

Super duplex stainless steel 2507 is welded by gas focusing plasma arc welding with or without nitrogen gas. The effects of nitrogen addition on the microstructure evolution and properties of SDSS 2507 weld joints are studied. The conclusions can be drawn as follows.

1. The addition of nitrogen increased the content of austenite in the WM from 22.2% to 40.2%. The optimal phase ratio can obtain SDSS 2507 welded joints with good quality and mechanical property. Thin grain boundary austenite and small intragranular austenite exist in the weld without nitrogen addition, while a large amount of coarse, side-plate Widmanstätten austenite appears in the weld with nitrogen addition.
2. The growth of ferrite in the WM exhibited a preferred orientation direction, while the orientation direction of the austenite phase was disordered. The max texture intensity of ferrite decreased when nitrogen was added.
3. The addition of nitrogen increased the microhardness of the austenite in the WM due to the solid solution strengthening of nitrogen and more dislocation tangle while it decreased the microhardness of ferrite.
4. The nitrogen, as the focusing gas, plays an important role in increasing the content of austenite. The influence of the nitrogen flow rate on the microstructure, mechanical property and corrosion resistance should be studied in gas focusing plasma arc welding of SDSS 2507.

## Figures and Tables

**Table 1:** Nomenclature.

Short Name	Full Name
SDSS	super duplex stainless steel
DSS	duplex stainless steel
WM	weld metal
FZ	fusion zone
HAZ	heat-affected zone
GTAW	gas tungsten arc welding
SMAW	shielded metal arc welding
GMAW	gas metal arc welding
FCAW	flux-cored arc welding
LBW	laser beam welding
EBW	electron beam welding
PAW	plasma arc welding
OM	optical microscope
XRD	X-ray diffraction
EBSD	electron backscatter diffraction
TEM	transmission electron microscopy

**Table 2:** Chemical composition of SDSS 2507 (wt. %).

C	Si	Mn	P	S	Ni	Cr	Mo	N	Cu	Fe
0.037	0.488	0.749	0.007	0.006	6.500	24.280	3.650	0.335	0.079	Base

**Table 3:** Welding parameters.

Welding Current (A)	Welding Speed (mm/min)	Plasma Gas Flow Rate (L/min)	Shielding Gas Flow Rate (L/min)	Back Shielding Gas Flow Rate (L/min)
100	350	1.2	25	10

**Table 4:** Chemical compositions of ferrite and austenite in different zones.

	Zone	Phase	Element (wt.%)				PREN
			Cr	Ni	Mo	N	
BM	_	Ferrite	28.366	5.267	3.846	0.050	41.858
		Austenite	26.854	7.230	2.488	0.520	43.384
Experiment A	WM	Ferrite	27.726	6.119	3.813	0.050	41.109
		Austenite	28.110	6.132	3.236	0.800	51.589
	HAZ	Ferrite	28.078	5.488	4.368	0.050	43.292
		Austenite	27.700	6.271	2.936	0.882	51.501
Experiment B	WM	Ferrite	27.909	5.887	3.740	0.050	41.051
		Austenite	28.163	6.158	3.281	0.745	50.910
	HAZ	Ferrite	28.266	5.504	4.192	0.050	42.900
		Austenite	27.746	6.803	2.922	0.692	48.461
	Austenite-enriched area	Ferrite	27.090	5.157	4.232	0.050	41.856
		Austenite	29.242	6.419	3.014	0.909	53.732

## Data Availability

The original contributions presented in the study are included in the article; further inquiries can be directed to the corresponding authors.
